# Frustration brake failure hypothesis of substance use disorders

**DOI:** 10.3389/fpsyt.2026.1896594

**Published:** 2026-08-14

**Authors:** Yorkiris Mármol Contreras, Rocio Sanchez Rivas, Shaina Banh, Iona C. Duncan, Sindhuja Earagolla, Thomas A. Green

**Affiliations:** 1 Department of Pharmacology and Toxicology, The University of Texas Medical Branch, Galveston, TX, United States; 2 Center for Addiction Sciences and Therapeutics, The University of Texas Medical Branch, Galveston, TX, United States; 3 John Sealy School of Medicine, The University of Texas Medical Branch, Galveston, TX, United States

**Keywords:** addiction, frustration, frustrative nonreward, incentive disengagement, relapse, substance use disorder

## Abstract

It is common knowledge that individuals with substance use disorders (SUDs) may persist seeking drug despite experiencing large degrees of frustration (i.e., due to loss of supplier, unsurmountable cost increases, etc.), beyond where one without an SUD would quit seeking. While preceding SUD hypotheses study various aspects of persistence of drug seeking in the face of negative consequences, this hypothesis posits a distinct mechanistic failure. We hypothesize that, unlike persons using drugs who do not have an SUD, a subset of persons with SUDs exhibit high motivation for drug specifically due to a failure in the ability for frustration to act as a brake on motivation. Frustration and motivation are intrinsically linked and share a biological interface which, if therapeutically targeted, could restore the ability for frustration to appropriately quell motivation and thus prevent some relapses. This paper outlines the existing and missing evidence required to support this hypothesis, with the hope that formalizing this hypothesis will spur important research in this area and yield new treatment possibilities for SUDs.

## 1 Introduction

Historically, a vast majority of research into substance use disorders (SUDs) has focused on incentive motivation and craving (1), impulsivity (2, 3), or habit (4). While each of these is important, we propose that frustration is a fourth facet of SUDs warranting attention.

Frustration is the negative emotional state produced when one’s will or goal is blocked unexpectedly (e.g. due to omission, reduction, delay, or physical blockage of an expected outcome) (5, 6). The utility of frustration is to quell motivation to prevent persistence in non-adaptive behavior (7). However, this function appears to fail in some individuals with SUDs, who may seek drug despite unreasonable surges in cost, loss of suppliers, or other frustrating events. Whereas persons without an SUD would eventually quit seeking due to high frustration, persons with an SUD may persist in seeking drug, often escalating to riskier behaviors. Unfortunately, little data exists on the frequency and extent to which individuals are undeterred by frustration when seeking drug. This limits our functional understanding of the underlying mechanism and hinders the development of pharmacotherapeutics or behavioral interventions targeting interactions between frustration and motivation.

**Figure 1:**
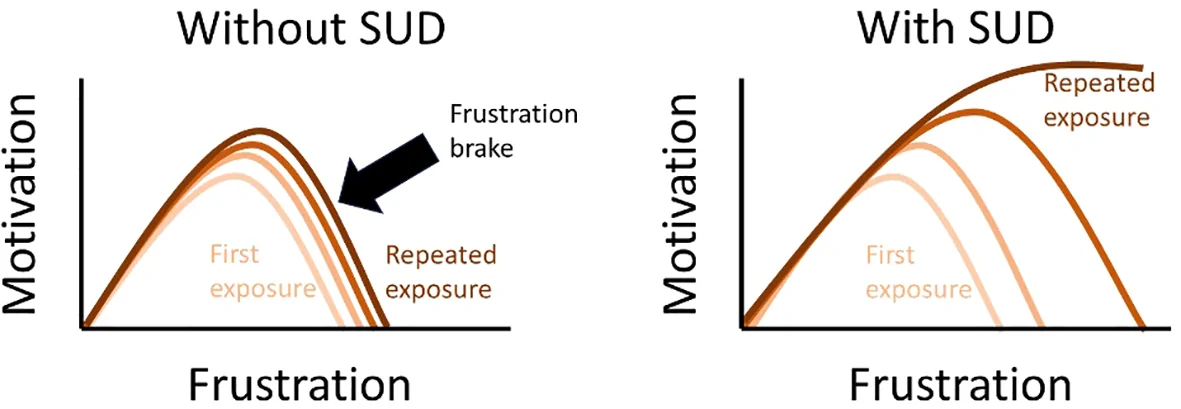
The relationship between frustration and motivation for drug. The left panel illustrates the fundamental relationship between frustration and motivation in individuals without an SUD when seeking drug: low levels of frustration increase motivation, whereas high levels of frustration decrease motivation (i.e. frustration brake). The right panel illustrates the relationship between frustration and motivation in some individuals with SUDs that develop frustration brake failure: in early stages of drug taking, high frustration decreases motivation for drug, but this function is weakened with repeated drug exposure, eventually leading to frustration-brake failure and persistent drug seeking despite any amount of frustration.

The frustration brake failure hypothesis of SUDs posits that in a subset of people suffering from SUDs, frustration fails to perform its necessary motivation-braking function (i.e., incentive disengagement), contributing to unrestricted drug-seeking (**Figure 1**). This results in a monophasic increase in motivation that persists despite high levels of frustration. Drug users without an SUD will eventually disengage as frustration escalates. This high motivation is not a function of general impaired executive control or negative reinforcement, rather a result of impaired frustration-related functions.

The novel therapeutic strategy arising from this hypothesis would **not** be to increase the *feeling* of frustration, but rather, to identify and target the neurobiological interface that typically enables frustration to modulate motivation, thereby rescuing the ability for frustration to *appropriately* attenuate motivation for drug during frustration. While previous literature has explored associations between frustration and SUDs (8, 9), specific deficits in frustration-attenuated motivation have not been explicitly considered or empirically investigated.

**Figure 1** depicts a theoretical framework of the hypothesis. In persons without an SUD, frustration maintains its biphasic function when seeking drug: low levels of frustration increase motivation while higher frustration levels decrease motivation (i.e., the frustration brake). In a subset of individuals who develop an SUD, repeated drug use alters this relationship such that frustration is unable to appropriately tame motivation, resulting in a monophasic function (i.e., brake failure).

## 2 Relevance to and distinction from existing hypotheses of SUDs

It is important to recognize the relevance of the current hypothesis to previously proposed frameworks of SUD pathogenesis.

### 2.1 Competing neurobehavioral decision systems

Bickel et al. (2016) proposed the CNDS hypothesis of cocaine addiction whereby individuals continuously choose to consume cocaine despite negative consequences due to a dysregulation of the competitive balance between impulsive and executive systems (3). According to the authors, the CNDS hypothesis is an extension of dual-systems theory that accounts for “self-control failure” (Bickel et al., 2016, p. 3). Therefore, the CNDS hypothesis is similar to the frustration brake failure hypothesis in that both recognize the role of altered communication between limbic and cortical processes in determining motivation for drug (impulsive choice vs. incentive disengagement). However, these hypotheses differ in key aspects of their proposed mechanisms and are likely to inform on different SUD populations. Whereas CNDS emphasizes the production of an impulsive phenotype: “a bias for immediate over delayed rewards” (3), the frustration-brake failure hypothesis focuses on persistent drug-seeking during frustrating situations. We hypothesize that individuals experiencing frustration brake failure persist in seeking drug regardless of delay, a significant departure from the framework proposed by Bickel and colleagues.

### 2.2 Incentive sensitization hypothesis

The Incentive Sensitization Hypothesis proposed by Robinson and Berridge in 1993, which posits that exposure to drugs of abuse heightens incentive motivation in susceptible populations (1, 10), is also highly relevant. Robinson and Berridge posit that “wanting” of drug (related to craving and motivation) increases with use while subjective “liking” may plateau or even decrease. While this hypothesis is well considered, it focuses on increased motivation alone and pays little attention to upstream regulators of motivation, such as frustration. While it is undeniable that motivation is high in many with SUDs, we propose malfunctioning frustration networks as a potential mechanism for how this aberrant motivation is expressed in some people.

The incentive sensitization hypothesis is credited with emphasizing the role of plasticity resulting from recurrent drug exposure in modifying motivation-driving circuitry, which we recognize within our hypothesis as a driver of frustration brake failure. However, increased “wanting” is not tied to a state of frustration in the Incentive Sensitization hypothesis; the brake failure hypothesis specifically deals with frustration encountered in drug seeking. In our view, the Incentive Sensitization hypothesis is useful, but incomplete. Only by supplementing the Incentive Sensitization hypothesis with hypotheses that focus on specific processes that fail to limit motivation could we greatly enhance opportunities for understanding addiction pathology and uncovering novel therapeutic strategies (i.e., restoring the ability for frustration to inhibit motivation).

### 2.3 Etiologic, theory-based, ontogenetic hierarchical hypothesis

While the cleverly named ETOH framework recognizes and emphasizes the role of the individual’s tendency toward negative emotionality (i.e. punishment sensitivity, negative expectancies) as a predisposing and contributing factor to SUD pathology (11), the current hypothesis is concerned with the progressive deterioration of the ability for a specific type of negative emotion, frustration, to redirect goal-oriented behavior.

## 3 Unique predictions of the frustration brake failure hypothesis

While the hypothesis is related to several existing theoretical frameworks, our hypothesis makes predictions not incongruent, but also not readily apparent from other hypotheses.

### 3.1 A specific, if as yet unknown, neurobiological target

This hypothesis, as described below, posits that there is a specific neurobiological substrate for frustration that acts to decrease motivation for reward. Because there is not a 1:1 correspondence between brain regions and functions, this neurobiological substrate will likely be at the circuit activity level (see below).

### 3.2 Persistence despite delay

To distinguish from the CNDS impulsivity hypothesis, ours predicts the persistence of drug seeking even as the reward is delayed.

### 3.3 Intact frustration perception that fails to decrease motivation

The crux of this hypothesis is that the frustration brake failure is not a lack of the aversive experience associated with frustration, but rather a functional decoupling of frustration from motivation.

### 3.4 Identification of a specific sub-population

We do not contend that every person with an SUD will show brake failure. For example, SUDs based purely on impulsivity would likely not have a brake failure. However, persons with SUD that exhibit brake failure may be effectively treated by targeting the frustration-motivation interface.

### 3.5 Behavioral markers

It is hypothesized that there will be human behavioral markers similar to barpress duration in rats that will be able to identify those with brake failure.

## 4 Distinction from existing hypotheses

It is important to note that this hypothesis is not in competition with the other discussed hypotheses. Rather, it focuses on a very specific aspect of SUDs: the frustration-motivation interface, and thus offers a novel, mechanistic explanation centered on the frustration-motivation interface. This unique focus is critical for inspiring research for novel therapeutic targets for this interface.

## 5 Relationship with fundamental constructs of SUDs

### 5.1 Compulsivity and impaired inhibitory control

While the frustration brake failure hypothesis accounts for increased drug seeking in a similar manner to compulsivity, habit formation, impaired cognitive flexibility, or general impaired executive control, this hypothesis focuses on a very specific mechanism–that of the frustration-motivation interface. Some might consider frustration brake failure a subset of impaired inhibitory control, but the neurobiology is likely distinct from other forms of executive function deficits.

### 5.2 Negative reinforcement and allostasis

Negative reinforcement and allostasis models explain drug seeking as a means to alleviate a negative internal state. While frustration is indeed a negative internal state warranting remediation, the drug seeking is not in itself an attempt to alleviate frustration, but rather a broken disengagement mechanism.

## 6 Existing and missing premise for the frustration brake failure hypothesis

Despite overwhelming anecdotal evidence for this hypothesis, there is yet little clinical research directly investigating the tenets underlying it. This may be due in part to the lack of scientific attention dedicated to understanding mechanisms of frustration in the last 50 years (5, 6). In addition to the significant challenges associated with empirically testing this hypothesis, many scientists already accept it as true, decreasing the perceived urgency of understanding the underlying mechanisms. Unfortunately, accepting this hypothesis as true without studying the precise mechanisms underlying this relationship yields the same result as not recognizing the relationship at all.

As mentioned, the purpose of this manuscript is to formalize this hypothesis and generate ideas that will spur the research necessary for scientific progress. This hypothesis provides four elements that should be empirically determined to better support therapeutic development. First, we must clarify the available evidence of the frustration brake in healthy individuals. Second, we must determine that frustration and motivation are separable but interacting constructs, given frustration’s direct modulation of motivated behavior. Third, there needs to be clinical evidence of frustration brake failure in SUD populations. Finally, it is important to determine the nature of the effect: does frustration brake failure for drugs result from a lack of the subjective feeling of frustration (i.e. not “feeling” frustrated despite facing obstacles), or does it result from an inability for frustration to quell motivation? To yield translational progress, we must identify pathways, neurobiological substrates, and behavioral practices that can modify the strength of frustration’s demotivating function and leverage these therapeutically.

**Table 1:** Criteria for the frustration brake failure hypothesis of SUDs.

Necessary clinical evidence to support the frustration brake failure hypothesis of SUDs
1. Frustration produces a biphasic effect on motivation. 2. Frustration and motivation have distinct but interacting neurocircuitry. 3. In some individuals with an SUD, recurrent drug use weakens the ability for frustration to decrease motivation for drug. 4. Individuals with an SUD who experience frustration brake failure still feel frustration (i.e., are not merely “insensitive”).

### 6.1 Frustration produces a biphasic effect on motivation

In simple terms, motivation is the willingness to do work to attain a goal, which is directly modulated by frustration, defined as the negative emotion associated with failure or difficulty in attaining a goal. As illustrated in the left panel of **Figure 1**, frustration has a biphasic effect on motivation, first increasing motivation before eventually decreasing it (i.e., frustration brake). For example, humans have been shown to exhibit a transient “burst” in motivated behavior when expected outcomes are blocked, sometimes known as an “extinction burst” (12, 13). This period of invigorated responding has been quantified in a variety of ways including increased rates of responding, faster response times, and more forceful responses (14–17). Supporting this, humans have self-reported increased levels of motivation concurrent with self-reported increased levels of frustration, decreased reaction times, and increased force of responding (18). When frustration persists, however, humans consistently suppress motivated behavior (19, 20), herein referred to as the frustration brake. Empirical demonstrations of this biphasic phenomenon have been reported in animal models since the 1920s (6, 17, 21, 22). Therefore, the widespread consensus in the field is that a conserved mechanism does indeed exist by which frustration situationally enhances and then brakes motivation. This part of the frustration brake failure hypothesis has the strongest evidence.

### 6.2 Frustration and motivation have distinct but interacting neurocircuitry

The neurobiological basis for the relationship between frustration and motivation is complex in that frustration cannot occur without motivation, and therefore, the neurocircuits controlling these functions are intrinsically linked (6). However, the fact that frustration can have biphasic effects on motivation suggest that the circuits controlling these functions likely diverge/converge to some degree. This is important for the therapeutic utility of the frustration brake hypothesis because without diverging or converging circuits, modulating frustration-related networks to produce demotivation would unavoidably result in the undesirable side effect of heightened feelings of frustration. We posit that one or more interfaces exist that modify the *strength* of the intrinsic link between frustration and motivation such that targeting these could restore the ability for frustration to decrease motivation for drugs without increasing subjective feelings of frustration.

#### 6.2.1 Motivation

Functional imaging studies have broadly elucidated the networks that control motivated behavior and frustration in humans. A common finding is that the mesolimbic reward system, which strengthens associations between behaviors and their respective outcomes, shows heighted activity during motivated behavior to facilitate learning, anticipation, and salience (i.e., “wanting”) (23–26). Indeed, heightened activity within the ventral striatum and the nucleus accumbens can predict relative motivation and motivational value of rewards (27–30). Moreover, activity within the prefrontal cortex, especially the orbitofrontal cortex (OFC) and ventromedial PFC (vmPFC), has been linked to aspects of motivation such as action selection, subjective valuation, and anticipation (23, 30–32). Additionally, several regions outside of the mesolimbic system are also highly implicated in motivation. For example, the insula shows heightened activity during motivational processes such as reward processing, anticipation, valuation, and effort investment (28–30, 33, 34). The hypothalamus modifies internal drives that contribute to motivation via its endocrine modulation of homeostatic states (35–38), and fMRI studies have confirmed this role (39). Though this is not an exhaustive list of motivation-related networks, these regions are essential to controlling motivated behaviors.

#### 6.2.2 Frustration

Studies where rewards are omitted or obstructed (i.e. frustration) report increased activity in the dorsal striatum, concurrent with decreased activity in the ventral striatum and ventromedial PFC (6, 40). Activity within the insula and lateral regions of the PFC are also reported during frustration (18, 41–43), specifically related to evaluating effort, outcomes, and uncertainty. Within the PFC, the anterior cingulate cortex (ACC) is especially involved in frustration due to its role in comparing outcomes to expectations and emotional regulation (40, 44–46), but activity within other prefrontal subregions such as the OFC is also modulated when rewards are omitted or uncertain (42, 43, 47). The habenula is highly implicated in frustration and is known to encode aversive outcomes by modulating dopaminergic networks (48, 49), and activity within this region is also reported in fMRI studies of frustration (42, 50, 51). Depending on the task, heightened activity within the amygdala is also reported (18, 42), usually regarded as specific to emotional stimuli.

Generally, mesolimbic regions that are activated by reward are deactivated under nonreward. Moreover, several cortical areas such as the PFC and insula are activated in both conditions, though for distinct functions, to diverging degrees, and with some topological segregation. The networks that control frustration and motivation are highly interconnected and in constant communication. Thus, sites where motivation- and frustration-related signals converge are a promising start to understanding the frustration brake. Indeed, our own research with rats confirms that the nucleus accumbens, a key integrative hub for motivation and frustration-related signals, regulates indices of both motivation and frustration-related behaviors (52). behavior

As stated above, the exact neurocircuitry mediating the frustration brake has yet to be discovered. However, because the mesolimbic dopamine system is so heavily involved with motivation, and frustration modifies motivation, the likely mechanism for the frustration brake is likely circuitry targeting the ventral tegmentum or the NAc. The input likely stems from other parts of the limbic circuitry such as the prefrontal cortex, amygdala, and/or habenula. While the exact circuitry is unknown, there is reason for confidence in the idea that frustration and motivation have different biological circuitry simply because the frustration-motivation relationship is biphasic.

### 6.3 In some individuals with an SUD, recurrent drug use weakens the ability for frustration to decrease motivation for drug

Research directly exploring whether frustration’s control over motivation is altered by SUDs does not exist, hence the purpose of this manuscript. Regardless, there is reasonable indirect and anecdotal evidence. First, there is extremely strong evidence for high motivation for drug in those with SUDs contributing to an inelastic demand for drug (53–60). Inelastic demand is a behavioral economics term for increased prices of a drug (i.e., frustration) that do not curb drug use. Our contention is that, in at least some with SUDs, this high motivation likely is a result of a failure of frustration to act as a brake on motivation.

Anecdotally, a subgroup of persons with SUDs sometimes resort to crime such as sex work or violence that are presumably beyond what most without an SUD would resort to for drug. For example, a 2017 report found that roughly 30% of property offenders in state prisons self-reported committing these offenses “to obtain money for drugs”, a statistic that has been replicated by multiple groups (61–63). It should be noted that this behavior is not exclusive to frustration brake failure, but to high motivation in general. If one makes a reasonable assumption that the frustration brake is what keeps non-SUD persons from engaging in sex work or violence to obtain drug, then it follows that the frustration brake must fail to curb the high motivation in some persons with SUDs. However, this idea needs to be confirmed scientifically. The important caveat that people can resort to risky behavior for a variety of psychological and psychosocial reasons. It should be investigated if persons who engage sex work or violence for drugs see it as an escalation of risky behavior as a last resort only when other “safer” methods of procuring drugs fail, or merely as an “easier” way to procure drugs. Indeed, reasons for turning to sex work or violence is not a simple decision as drug can lower inhibitions or act as a self-medication. Additionally, it would be important to compare the incidence of sex work or violence specifically to procure drugs in non-SUD drug users vs. those with SUDs. Frustration itself can lead to aggression (40, 64), further complicating the situation. Because of the speculative nature of this condition, these caveats can and need to be addressed by studies on humans specifically teasing apart these constructs. Despite much anecdotal evidence, lack of scientific clinical evidence for this represents the weakest part of the frustration brake failure hypothesis.

### 6.4 Individuals with an SUD who experience frustration brake failure still feel frustration (i.e., are not “insensitive”)

There are two possible explanations for how frustration could fail to attenuate motivation in people with an SUD: either people with SUDs are largely insensitive to frustration, or they feel frustration but the neurobiological link (i.e., brake) between frustration and motivation is weakened. Though it should be thoroughly investigated, evidence supports the latter assertion. Multiple studies have reported that coping with frustration is a common cause of relapse, underlying up to 25% of relapses associated with negative emotions (9, 65–67). Moreover, heightened emotional reactivity is both a risk factor for and result of SUDs (8, 68). Therefore, we infer that individuals with SUDs generally do experience frustration, providing good evidence for this assertion. However, as with all of the hypothesis, more human data would solidify the assertion.

## 7 What basic science can contribute to testing the hypothesis

Basic science has obvious advantages and disadvantages that complement clinical science, leading to the translational approach necessary for significant progress in therapeutic development. To facilitate this, analogous methods that can be used in human as well as animal research will be crucial in improving the animal to human pipeline of therapeutic development.

To this end, we recently developed and validated a way to assess frustration-related behavior with motivation in rats by monitoring bar press durations (frustration) in addition to number (motivation) of bar presses during standard operant motivation procedures (69–71). Our work shows a replicable spike in frustration-related bar press durations for fentanyl or cocaine infusions shortly before the rats extinguish responding under a progressive ratio procedure (69, 70), consistent with frustration acting as a brake on motivation. Simultaneously measuring frustration and motivation opens new opportunities for directly interrogating the fundamental relationship between these constructs. Combining this tool with cutting-edge technologies such as fiber photometry or *in-vivo* single cell imaging, which is complementary to human neuroimaging, will allow for the assessment of the neurocircuitry underlying the frustration-motivation interface during behavior. Furthermore, chemo- and optogenetic applications can be used in rodents to manipulate circuit activity, which could provide further causal data to test this hypothesis.

## 8 Future directions

The most urgent need for the adequate testing of this hypothesis is clinical assessment of frustration specifically as it relates to SUDs and more specifically as it relates to frustrations in procuring drug. Rather than comparing individuals with an SUD to non-drug users (who obviously do not seek drug), the assessment needs to compare persons with SUDs to non-SUD persons using drugs. This would require a clear distinction between those with an SUD to matched non-SUD drug users. The heterogeneity of presentation of SUDs, the difficulty in diagnosis, and the subjective nature of DSM criteria likely means DSM criteria for SUDs alone may not be great at distinguishing these populations.

### 8.1 Specific experimental techniques

A stepwise strategy could provide important rigorous results. First, open-ended interviews with people with SUDs could provide critical information on previously unappreciated factors influencing drug use. This information could then be used to develop an Ecological Momentary Assessment (EMA) enabling subjects to answer relevant questions on their smart phone in their natural environment related to frustration in procuring drugs. This will satisfy Criteria 1 (biphasic effects), 3 (weakened brake in SUDs), and 4 (still feel frustration). Information from this could be used to develop a structured interview to assess validated and standardized questions to stratify subjects and measure the frustration brake in non-SUD vs. SUD drug users, adding further evidence to those same criteria. Additionally, the questionnaires could be used in conjunction with fMRI brain imaging of subjects to gain information on Criteria 2 (circuitry). Another strategy is to develop and validate a computerized or operant task that evaluates the strength of the frustration-motivation interface. This task could be used to identify persons with SUD that have a weakened interface, to determine how SUD severity modifies the interface, and could be applied prospectively to track the development of brake failure among drug users. The second most immediate need is to better align clinical and preclinical research for translation. To this end, we have developed a point subtraction task in rats for sucrose pellets that has proven useful in predicting fentanyl seeking (i.e., motivation) before the rats are ever exposed to drug. We found that rats that exhibit heightened frustration during point subtraction for sucrose demonstrated increased motivation for fentanyl in the future (71). In this case, frustration was determined by increases in barpress durations, but we have also found that the same point subtraction task increases force of responding (unpublished data). Several human studies find that subjects hit a button with more force when they are more frustrated (41, 72–74), offering some convergence that can be used to create the basis of a translational pipeline.

## 9 Summary and conclusions

This is a curious case where most of the field already believes that frustration fails to appropriately curb motivation in some individuals with SUDs, meaning that this is a very plausible hypothesis despite it not being explicitly articulated or thoroughly investigated. The purpose of this publication is to motivate clinical and basic scientists to rigorously test the hypothesis, thereby advancing the field and generating new therapeutic avenues to restore the checks and balances of the brain’s reward system. Despite evidence in support of the frustration brake failure hypothesis of SUDs, numerous gaps in the literature are yet to be addressed.

It should be noted that there is considerable heterogeneity in SUDs and there are likely many subtypes of SUDs. As such, the frustration brake failure hypothesis will only be relevant to a subset of people with SUDs. Additionally, there are considerable differences in how different drugs modify neurobiology. We do not contend that tobacco and alcohol engender the same frustration brake failure as cocaine or heroin. Along the same lines, the frustration brake failure should not be equal across all phases of addiction.

Lastly, it is important to consider causality: is frustration brake failure a predisposing factor for SUDs or a consequence of drug use? Our model assumes the frustration brake failure is a response to drug use, as people do not have extremely high motivation for a drug they have never experienced, and there is no evidence for a lack of a frustration brake prior to drug use. However, it is also conceivable that individuals with a naturally weaker frustration-motivation interface will be at a higher risk of developing brake failure for a particular drug. As with all assumptions, this should be determined empirically.

The frustration brake failure hypothesis provides a novel approach to the study of SUD relapse, particularly the ability (or lack thereof) to disengage from drug seeking in the face of frustration. It simultaneously acts as an organizing framework, a general roadmap to a novel therapeutic strategy, and a call to arms for clinicians to test the hypothesis. This hypothesis differs from other hypotheses in that it focuses specifically on the frustration-motivation interface during SUD relapse rather than motivation, habit, or impulsivity alone. Although none of the 4 criteria are fully satisfied in humans at this point, key bits of evidence from the literature are supplemented with overwhelming anecdotal evidence to provide the necessary premise for the hypothesis.

## Data Availability

The original contributions presented in the study are included in the article/supplementary material. Further inquiries can be directed to the corresponding author.
